# Evaluation of hepatic injury in chronic hepatitis B and C using APRI and FIB-4 indices compared to fibroscan results

**DOI:** 10.1097/MS9.0000000000002095

**Published:** 2024-05-15

**Authors:** Narges Najafi, Alireza Razavi, Hamed Jafarpour, Maedeh Raei, Zahra Azizi, Lotfollah Davoodi, Amirsaleh Abdollahi, Mehran Frouzanian

**Affiliations:** a Department of Infectious Diseases, School of Medicine, Antimicrobial Resistance Research Center, Communicable Diseases Research Institutes, Qaem Shahr Razi Hospital, Mazandaran University of Medical Sciences; b Student Research Committee, School of Medicine, Mazandaran University of Medical Sciences; c Gastrointestinal Cancer Research Center, Non-Communicable Diseases Institute, Mazandaran University of Medical Sciences, Sari, Iran

**Keywords:** APRI, FIB-4, fIBROSCAN, HBV, HCV, hepatic injury

## Abstract

**Background:**

Hepatitis B (HBV) and hepatitis C viruses (HCV) are significant causes of liver disease worldwide. Liver fibrosis (LF) is a complication of chronic liver damage caused by HBV and HCV due to our limited knowledge comparing the diagnostic performance of platelet to aspartate aminotransferase ratio index (APRI) and fibrosis-4 (FIB-4) index with fibroscan.

**Methods:**

This study evaluated liver damage in HBV and HCV using APRI, FIB-4, and fibroscan indices. This retrospective cohort descriptive-analytical study was conducted on patients with HBV and HCV. This study uses laboratory results and imaging to investigate liver damage in chronic HBV and HCV patients. APRI and FIB-4 were computed based on laboratory results.

**Results:**

A total of 185 patients (82 hepatitis B and 103 hepatitis C) were included in the study. Thirteen patients had liver cirrhosis. There was no statistically significant difference between the fibroscan results in the two groups (*P*=0.99). The HBV group’s mean APRI and FIB-4 were lower than HCV, but no significant difference was observed (*P*>0.05). Our results in HBV and HCV patients showed that APRI and FIB-4 accomplished well anticipating cirrhosis with an area under the receiver operating characteristic curve (AUC) of 0.771–0.845 and 0.871–0.910, respectively.

**Conclusion:**

Fibroscan is a powerful tool superior to APRI and FIB-4 in predicting LF and cirrhosis. Nevertheless, APRI and FIB-4 are inexpensive and non-invasive indicators with acceptable efficacy in predicting advanced fibrosis or cirrhosis. However, these two measures are not reliable in low-grade fibrosis.

## Introduction

Highlights

- Drug addiction injection is the top risk factor for chronic hepatitis B and C.
- Most patients had mild to no liver scarring; however, a few had cirrhosis.
- APRI and FIB-4 scores accurately detect cirrhosis in hepatitis B and C, with varying cut-off values.
- Regular monitoring and early detection are vital for liver damage in chronic hepatitis B and C.

Viral hepatitis is one of the eminent causes of liver disease worldwide. About 400 million people live with the hepatitis B virus (HBV) or hepatitis C virus (HCV) worldwide, of which 1.4 million die each year from complications^1,2^. Complications of chronic liver damage from HBV and HCV include liver fibrosis (LF), which can lead to cirrhosis, hepatic failure, and hepatocellular carcinoma (HCC). Fibrotic changes in the liver are part of functional and structural variations in chronic liver diseases (CLDs). Accurate estimation of the progression of LF in surveillance prognosis, treatment, and indication for treatment initiation are substantial in these patients^3–6^. In recent decades, liver biopsy has been considered the gold standard in determining the staging of LF. However, this invasive process has several disadvantages, including sampling error and some risks for patients^7^. Because of these complications and the error of liver biopsy, researchers are looking for new non-invasive parameters, indices, and tests to assess the severity of fibrosis and the degree of inflammation^8^. Several alternative non-invasive methods have been developed and improved the evaluation of LF stages, such as the fibrosis-4 (FIB-4) index, transient elastography ultrasound (Fibroscan), and platelet to aspartate aminotransferase ratio index (APRI)^9–13^. APRI and FIB-4 are recommended as non-invasive methods to designate the LF stages in countries with limited resources by the WHO and several guidelines^13–17^. Also, FIB-4 and APRI scores were confirmed in some specific groups of patients and had a good relationship with liver biopsy^18–21^. However, in the latest non-invasive methods, liver stiffness measurement (LSM) using fibroscan has become the most prevalent non-invasive method for assessing LF in many countries^22,23^. Fibroscan is widely used due to its reproducibility and superb inter-and intra-observer agreement and has been validated in several studies that have shown an excellent correlation to histological assessment by liver biopsy^24–26^. However, our knowledge of comparing the diagnostic performance of APRI and FIB-4 compared with fibroscan is limited. This study aims to evaluate the extent of liver damage in chronic hepatitis B and C using APRI and FIB-4 indices and compare it with the results of a fibroscan.

## Material and methods

### Study design and population

This retrospective cohort descriptive-analytical study was performed on patients with chronic hepatitis B and C accompanied by liver injury. This study investigates liver damage in patients with chronic hepatitis B and C using their laboratory results and imaging. This study was conducted by the STROCSS criteria^27^. This study was conducted at Mazandaran University of Medical Sciences after the approval of the ethics committee in biomedical research with the code (IR.MAZUMS.REC.95.2426).

### Data collection and sample size

All records of patients referred to the clinic until 2020 were evaluated, and patients with positive HBsAg and HCV-Ab by enzyme‑linked immunosorbent assay (ELISA) or hepatitis nucleic acid using the polymerase chain reaction (PCR) tests and a history of liver fibroscan examinations were elected. The study excluded patients with concurrent liver conditions such as autoimmune hepatitis, fatty liver, and Wilson’s disease. The medical students retrieved data from a checklist that comprised two sections: one containing demographic information and the other containing clinical information. The demographic section had details such as age, sex, and the type of hepatitis (HCV or HBV), as well as laboratory biomarkers like platelet count (PLT), aspartate aminotransferase (AST), and alanine aminotransferase (ALT). Additionally, the checklist included the results of liver fibroscan obtained using the FibroTouch 502 device (Echosens). According to the METAVIR scoring system, fibrosis severity is classified into five levels: fibrosis score F0–F1: no liver scarring or mild liver scarring, fibrosis score F2: moderate liver scarring, fibrosis score F3: severe liver scarring, fibrosis score F4: advanced liver scarring (cirrhosis)^28^.

### Data analysis

Based on the extracted information, APRI and FIB-4 index were computed according to the following formulas:


$$\mathrm{APRI}=(\frac{\frac{\mathrm{AST Level}\;\left(\frac{\mathrm{IU}}{L}\right)}{\mathrm{AST}\;\left(\mathrm{Upper Limit of Normal}\right)\;\left(\frac{\mathrm{IU}}{L}\right)}}{\mathrm{Platelet count}\;\left(\frac{10^{9}}{L}\right)})\times 100$$

$$\mathrm{FIB}-4=\frac{\mathrm{Age}\;\left(\mathrm{years}\right)\times \mathrm{AST Level}\;(\frac{U}{L})}{\mathrm{Platelet count}\;\left(\frac{10^{9}}{L}\right)\times \sqrt{\mathrm{ALT}\left(\frac{U}{L}\right)}}$$

First, the Kolmogorov–Smirnov test was utilized to evaluate the normality of the data. Qualitative variables based on frequency (percentage) and quantitative variables based on mean±standard deviation (SD) were presented. The χ^2^ test and independent T-test were used to analyze the data. The receiver operating characteristic (ROC) curve and area under the ROC curve (AUC) were operated to assess the sensor’s validity and determine the desired point for laryngoscopy stiffness. A logistic regression analysis was carried out to determine the cut-off points of FIB-4 and APRI for HBV, and HCV. For data analysis, the SPSS software package (version 22.0, SPSS Inc.) was used at a significance level of 0.05.

## Results

**Figure 1:**
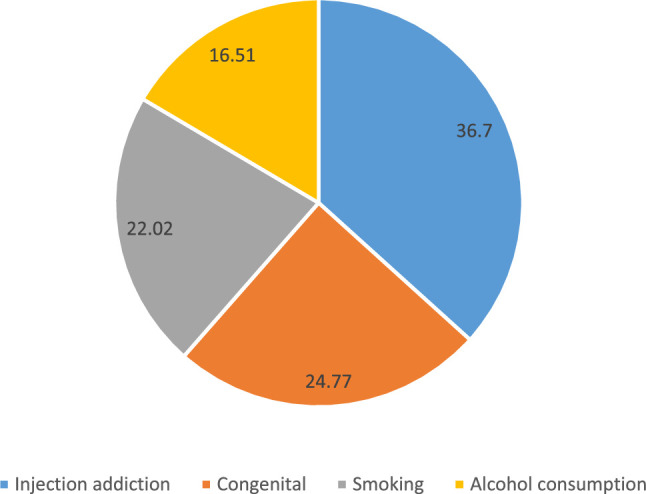
Percentage of risk factors of patients with hepatitis.

A total of 450 patients were checked, and 185 patients (82 hepatitis B and 103 hepatitis C) were included in the study based on the eligibility criteria. The mean age of patients was 38.85±13.60 years, of which 98 (53%) were male. General characteristics of the study participants are shown in Table 1. One hundred nine patients had risk factors, the highest risk factor being injecting drug addiction (36.7%) (Fig. 1). There was no statistically significant difference between gender and risk factors in hepatitis B and C groups (*P*>0.05, Fig. 1).

**Table 1:** General characteristics of the study participants

Variables	Values
Age (year) mean±SD	38.85±13.6
Sex, *n* (%)	
Male	98 (53)
Female	87 (47)
AST (U/l) mean±SD	52.64±24.32
ALT (U/l) mean±SD	54.18±31.53
Platelet count (10^9^/l) mean±SD	194.41±92.73
FibroScan results	Number (percent), *n* (%)
F0–F1 (<kPa)	154 (83.24)
F2 (7–8.99 kPa)	2 (1.08)
F3 (9–12.49 kPa)	18 (9.72)
F4 (≥12.5 kPa)	11 (5.94)

ALT, alanine aminotransferase; AST, aspartate aminotransferase.

Liver ultrasound results demonstrated that 13 patients had liver cirrhosis, the frequency of liver cirrhosis was higher in hepatitis C patients, and there was no significant difference between the types of hepatitis (*P*=0.89). The fibroscan results are shown in Table 2; most patients (83.24%) had F0 and F1. In both groups, one patient was F2, and 15.68% of patients were F3 and F4. There was no statistically significant difference between the fibroscan results in the HBV and HCV groups (*P*=0.99) (Table 2).

**Table 2:** Frequency (%) of ultrasound and fibroscan results

Variables	HBV (*N*=82)	HCV (*N*=103)	P*
Cirrhosis, *N* (%)**			
No	76 (92.68)	96 (93.20)	0.89
Yes	6 (7.32)	7 (6.80)	
Fibroscan, *N* (%)			
Mild			
F0	49 (59.76)	63 (61.17)	0.99
F1	19 (23.17)	23 (22.33)	
Moderate			
F2	1 (1.22)	1 (0.97)	
Severe			
F3	8 (9.76)	10 (9.71)	
Cirrhosis			
F4	5 (6.10)	6 (5.83)	

* Chi-Square

** Cirrhosis based on ultrasound

HBV, hepatitis B virus; HCV, HBV, hepatitis C virus.

Table 3 shows the mean and SD of patients’ APRI and FIB-4 scores by type of hepatitis. The HBV group’s mean APRI and FIB-4 scores were lower than HCV, and no significant difference was observed (*P*>0.05) (Table 3).

**Table 3:** Mean and standard deviation of APRI and FIB-4 indices in two groups.

Variables	HBV (*N*=82)	HCV (*N*=103)	P*
APRI, *N* (%)	0.81 (0.78)	1.03 (1.02)	0.123
FIB-4, *N* (%)	1.25 (0.88)	1.37 (1.00)	0.414

* Independent T-Test

APRI, aspartate aminotransferase ratio index; FIB-4, fibrosis-4; HBV, hepatitis B virus; HCV, hepatitis C virus.

Table 4 shows the mean and SD of APRI and FIB-4 scores of patients with F2 and higher fibroscan scores by hepatitis type. The HBV group’s mean APRI and FIB-4 scores were lower than HCV, and no significant difference was observed (*P*>0.05) (Table 4).

**Table 4:** Mean and standard deviation of APRI and FIB-4 indices in patients with F2 and higher fibroscan scores in two groups.

Variables	HBV (*N*=14)	HCV (*N*=17)	P*
APRI, *N* (%)	1.54 (1.26)	2.23 (1.77)	0.233
FIB-4, *N* (%)	2.22 (1.26)	2.53 (1.55)	0.543

* Independent T-Test

APRI, aspartate aminotransferase ratio index; FIB-4, fibrosis-4; HBV, hepatitis B virus; HCV, hepatitis C virus.

**Figure 2:**
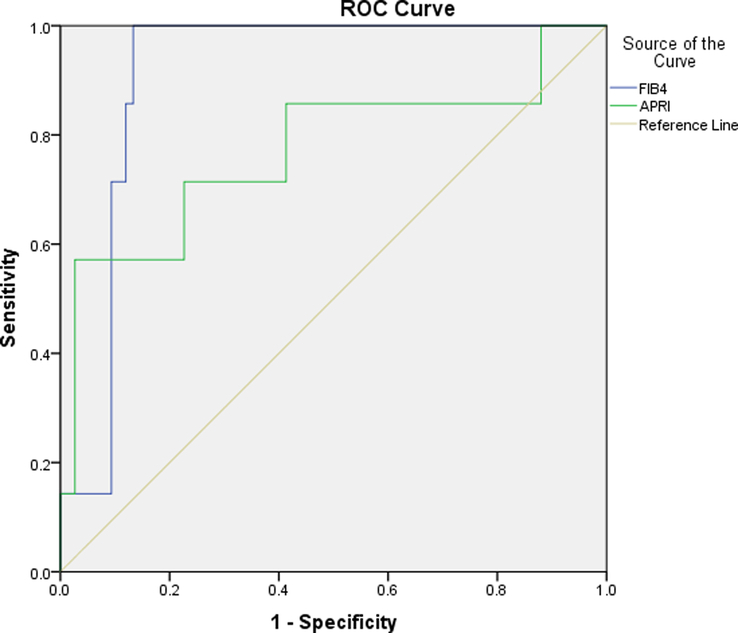
The ROC curve of FIB-4 and APRI in HBV group. APRI, aspartate aminotransferase ratio index; FIB-4, fibrosis-4; HBV, hepatitis B virus; ROC, receiver operating characteristic.

**Figure 3:**
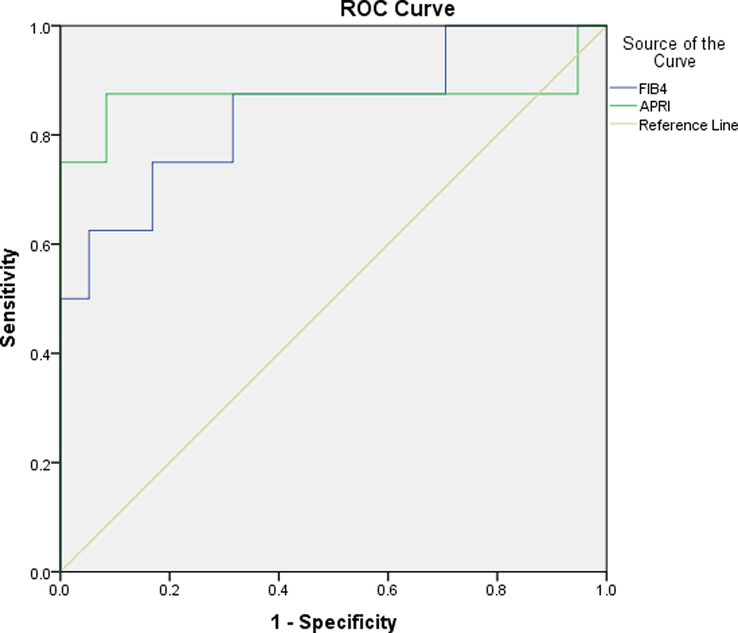
The ROC curve of FIB-4 and APRI in HCV group. APRI, aspartate aminotransferase ratio index; FIB-4, fibrosis-4; HCV, hepatitis C virus; ROC, receiver operating characteristic.

Since in this study, the results of fibroscan were considered the gold standard for cirrhosis, in HBV and HCV groups, 5 and 6 individuals had cirrhosis, respectively. The AUC for APRI in the HBV and HCV groups is between 0.7 and 0.9, which indicates that APRI is a good measure of cirrhosis (Table 5, Fig. 2). APRI greater than 1.07 had a sensitivity of 71.4% and a specificity of 77.3% in the HBV group. Moreover, among HBV patients, APRI positive and negative predictive values (PPV and NPV) were 22.73% and 96.67%, respectively. APRI greater than 1.8 sensitivity and specificity in the HCV group were 87.5% and 91.6%, respectively. Also, in these patients, the PPV and NPV of APRI were 42.86% and 98.88%, respectively. The AUC of FIB-4 was between 0.8-0.9 in both groups, which signifies that the FIB-4 is an accurate measure (Table 5, Figs. 2 & 3). In both groups, FIB-4 greater than 1.9 sensitivity and specificity were 85.7% and 86.7%, respectively (Figs. 2 & 3). Moreover, in the HBV group, the PPV and NPV of FIB-4 were 37.5% and 98.5%, respectively. The PPV and NPV of FIB-4 were 31.58% and 98.81%, respectively, in the HCV group. Fibroscan results for the study showed that most patients had F0 and F1 scores, and there was no significant difference in outcomes between the HBV and HCV groups. The APRI and FIB-4 scores were also reported and found to be accurate measures of cirrhosis in both groups.

**Table 5:** AUC of APRI and FIB-4 indices in two groups.

Groups	Indices	AUC (95% CI)	*P*
HBV	APRI	0.771 (0.544–0.998)	0.018
	FIB-4	0.910 (0.846–0.975)	0.000
HCV	APRI	0.845 (0.680–1.000)	0.001
	FIB-4	0.871 (0.655–1.000)	0.001

APRI, aspartate aminotransferase ratio index; AUC, area under the receiver operating characteristic curve; FIB-4, fibrosis-4; HBV, hepatitis B virus; HCV, hepatitis C virus.

## Discussion

This study was designed to evaluate the measure of liver damage in chronic hepatitis B and C using APRI and FIB-4 and compared to fibroscan results. We found that APRI greater than 1.07 predicted cirrhosis with more than 70% sensitivity and specificity in HBV patients. PPV and NPV of APRI in HBV patients were 22.73% and 96.67%, respectively. In the HCV group, at a cut-off level greater than 1.8, the sensitivity and specificity of APRI in predicting cirrhosis were 90% approximately, with PPV of 42.86% and NPV of 98.88%. In this descriptive-analytical retrospective study, FIB-4 greater than 1.9 could predict cirrhosis development in both groups with a sensitivity and specificity of nearly 86%. In patients with HBV, PPV and NPV of FIB-4 were 37.5% and 98.5%, respectively. The exact values for the HCV group were 31.58% and 98.81%, respectively. Our results in HBV and HCV patients showed that APRI and FIB-4 accomplished well anticipating cirrhosis with an AUC of 0.771–0.845 and 0.871–0.910, respectively.

Over the years, non-invasive methods for assessing LF due to HBV and HCV have been offered. The APRI and FIB-4 index are the most potent implements among these diagnostic tests^29^. The FIB-4 index is a non-invasive method for assessing LF that is calculated based on variables such as age, aspartate aminotransferase (AST), alanine aminotransferase (ALT), and platelet count. This index was first presented by AIDS researchers Pegasys Ribavirin International Coinfection Trial (APRICOT) study to assess the presence of LF in HCV patients coinfected with human immunodeficiency virus (HIV)^30^. In the present study, among HBV patients with an F2 score or higher, the mean FIB-4 scores, as a function of the severity of liver fibrosis, was higher than the mean FIB-4 score in all HBV patients (2.22±1.26 vs. 1.25±0.88). Moreover, in the HCV group, the mean scores of FIB-4 in patients with an F2 score or higher were more significant than the mean scores of the entire HCV group (2.53±1.55 vs. 1.37±1.00). The mean APRI ratio of AST to platelet was 0.81±0.78 and 1.03±1.02 in the HBV and HCV groups, respectively. These values were higher in people with an F2 score or higher in both groups. However, although the HCV group’s mean FIB-4 and APRI scores were higher than the HBV group, no significant difference was observed. APRI and FIB-4 may be affected by different factors, such as age and severity of liver inflammation^31^. The lower age and severity of inflammation in our study resulted in lower APRI and FIB-4 scores than the results reported by some surveys^32^.

Our findings revealed that APRI and FIB-4 have less ability to diagnose low-grade fibrosis than severe fibrosis. Previous studies have shown that the FIB-4 index could not significantly distinguish the severity of hepatic fibrosis in group F0 from the higher fibrosis score, F1, and F2 from F3^20,29^. Some studies on patients with chronic HBV in China showed that APRI was not significantly different from stage F1 with F2 and stage F3 with F4^18,31^.

Generally, at AUC less than 0.7, the detection accuracy is poor and unreliable; at 0.7 ≤AUC ≤0.9, the detection accuracy is satisfactory, and at 0.9 < AUC ≤1, the detection accuracy is excellent^33^. Sensitivity and specificity above 80% in LF measurement methods are appropriate^31^. A cohort study aimed to evaluate LF with four non-invasive instruments containing APRI and FIB-4 in Asian patients with chronic viral hepatitis demonstrated that the APRI and FIB-4 AUCs were 0.73 and 0.61, respectively^34^. In a study of 1543 patients with HBV in China, APRI and FIB-4 predicted cirrhosis with AUCs of 0.71 and 0.79, respectively^35^. Our findings revealed that APRI and FIB-4 AUCs were 0.771 and 0.910 in HBV patients, respectively, and 0.845 and 0.871 in HCV patients. However, a separate meta-analysis showed that APRI and FIB-4 could detect LF with moderate sensitivity and accuracy in patients with chronic hepatitis B and are not absolute alternatives to liver biopsy^36^. Since fibroscan showed a better potency to differentiate LF stages than APRI and FIB-4^37^, the capability of APRI and FIB-4 to detect LF stages was examined compared to the results of fibroscan in our study. Our results demonstrated that APRI and FIB-4, with good sensitivity and accuracy, can only diagnose severe fibrosis and cirrhosis and are incapable in the early stages of fibrosis.

Recently, a cross-sectional multicenter study of 2000 patients with chronic HCV showed that APRI greater than or equal to 1 yielded a sensitivity of 70.1% and a specificity of 80.6% with an AUC of 0.834 for cirrhosis. Specificity increased to 96.3% at the cut-off level of APRI greater than or equal to 2. The cut-off level of FIB-4 greater than or equal to 1.45, and the sensitivity, specificity, and AUC were 52.4%, 91.0%, and 0.829, respectively. In significant fibrosis, APRI accomplished better than FIB-4 (AUC: 0.84 vs. 0.80, *P*<0.001). APRI greater than or equal to 0.5 and FIB-4 greater than or equal to 1.45 showed a sensitivity of 82.3% and 74.4% and a specificity of 65.4% and 69.8%, respectively, in predicting significant fibrosis. APRI greater than or equal to 2 and FIB-4 greater than or equal to 3.25 had a PPV of 86.9% and 80.4%, respectively, for predicting cirrhosis^38^.

In our findings, in the HCV group, APRI greater than 1.8 resulted in a sensitivity and specificity of nearly 90% with a PPV of 42.86%. FIB-4 greater than 1.9 yielded sensitivity and specificity slightly less than 90% with a PPV of 31.58%. However, more than 98% NPV in both APRI and FIB-4 in HCV patients means that a significant proportion of patients can be easily managed. Our results demonstrated that the sensitivity and specificity of APRI in the HCV group with the cut-off level greater than 1.8 were more than in the HBV group with APRI greater than 1.07. APRI seems to be more accurate in diagnosing HCV-induced LF^39^. However, there was no significant difference in the sensitivity and specificity of FIB-4 between the two groups, although some studies have shown otherwise^39^. More well-defined and extensive studies are required to investigate the accuracy of APRI and FIB-4 compared to fibroscan.

There have been several limitations in the current study. First, patients were enrolled in a study from a referral center that could cause selection bias. Second, we used fibroscan as our standard reference, while the gold standard is a liver biopsy. Thus, there was a major obstacle to appraising inter-and intra-observer variation. However, significant studies have shown a good association between fibroscan and histologic evaluation with liver biopsy^24,26^. Third, due to retrospective data collection, some data were missed, such as a history of drugs affecting ALT levels. Finally, due to the small sample size of the current study, the number of patients with moderate and severe fibrosis was small. Therefore, the comparison with FO and F1 groups was not possible.

## Conclusion

Fibroscan is a reliable tool for predicting LF and cirrhosis in patients with chronic hepatitis, which is superior to APRI and FIB-4. However, APRI and FIB-4 are inexpensive, non-invasive indicators for predicting the possibility of advanced fibrosis or cirrhosis in patients with chronic hepatitis but are unreliable in low-grade fibrosis.

## Ethical approval

This study was approved by the Ethical Board of Mazandaran University of Medical Sciences (IR.MAZUMS.REC.95.2426).

## Consent

Patient consented was not mandated for this retrospective observational study.

## Sources of funding

We did not receive any funds for this research.

## Author contribution

A.R., A.A., H.J., Z.A. and M.F. participated manuscript preparation. L.D., N.N., M.R. and A.R. participated in data curation and manuscript preparation and design. A.R. and M.R. conducted the statistical analysis and reviewed the manuscript. All authors had access to the data and take responsibility for the integrity of data and the accuracy of data analysis. All authors have read and approved the manuscript.

## Conflicts of interest disclosure

The authors declare no conflicts of interest.

## Research registration unique identifying number (UIN)

This study was registered with the code (IR.MAZUMS.REC.95.2426) at Mazandaran University of Medical Sciences after the approval of the ethics committee in biomedical research.

## Guarantor

Lotfollah Davoodi.

## Data availability statement

Data are available upon reasonable request. All data generated or analyzed during this study are included in this article.

## Provenance and peer review

Not commissioned, externally peer-reviewed.

## Permission to reproduce material from other sources

No material from other sources is included in this study
